# Data on antimicrobial, barrier, and mechanical properties of biocomposites prepared from carrot pomace and wheat gluten with varied eugenol content

**DOI:** 10.1016/j.dib.2024.110490

**Published:** 2024-04-30

**Authors:** Danila Merino, Paolo Bellassi, Lorenzo Morelli, Athanassia Athanassiou

**Affiliations:** a Smart Materials, Istituto Italiano di Tecnologia, Genoa, Italy; b Department for Sustainable Food Process—DiSTAS, Università Cattolica del Sacro Cuore, Via Bissolati 4, 26100 Cremona, Italy

**Keywords:** Plant biomass, Wheat gluten, *Escherichia coli*, *Staphylococcus aureus*, Bioplastics, Water vapor permeability, Mechanical properties

## Abstract

This article presents analyzed data on the antimicrobial, barrier, and mechanical properties inherent to films created by blending carrot pomace with wheat gluten and polyglycerol-3 plasticizer and combined with varying contents (0 wt.%, 3 wt.%, and 5 wt.%) of eugenol, a natural antimicrobial compound derived from essential oils. The integration of carrot pomace, wheat gluten, plasticizer, and eugenol involved meticulous mortar and pestle processing, ensuring a homogenous blend. Subsequently, the mixture was compression-molded in a hydraulic press to fabricate the films. Standard bacteria strains—*Escherichia coli* ATCC 25922 and *Staphylococcus aureus* ATCC 6538—are used in the antimicrobial evaluation, and antimicrobial efficacy is measured using OD_600_ measurements. Water vapor permeability (WVP) measurement effectively defines the films' potential to prevent water vapor infiltration. Mechanical properties are assessed by determining elastic modulus, tensile strength, and elongation at break, which together reveal the films' adaptive flexibility and durability. The dataset presented herein holds substantial promise for food packaging applications. Researchers in the food packaging industry can leverage the antimicrobial and barrier property data to design novel packaging materials, potentially enhancing shelf-life and food safety. Engineers and material scientists can utilize the mechanical properties data to develop structurally robust and flexible materials.

Specifications Table

**Table:** 

Subject	Materials science
Specific subject area	Polymers and plastics; material characterization, biomaterials, materials application
Data format	Raw, Analyzed
Type of data	Graph, Figure
Data collection	Antimicrobial Assays: *Staphylococcus aureus* ATCC 6538 and *E. coli* ATCC 25922 incubated with films. Film samples UV-sterilized for 30 min per face. Microbial growth monitored at 37 °C by OD_600_ for 28 h after 12 h pre-incubation at 4 °C. Samples analyzed in triplicate. Water Vapor Permeability: Aluminum-based capsules filled with 300 µL water (100 % RH) sealed with biocomposite samples. Capsules stored at 0 % RH with silica gel. Weight loss monitored over time. Samples analyzed in triplicate. Mechanical Properties: INSTRON 3365. Samples conditioned in a Espec SH-262 and cut into a dog-bone shape. Thickness measured, 4 specimens tested per sample at 3 mm/min pace. All results shown as average ± SD.
Data source location	Istituto Italiano di Tecnologia (IIT), Via Morego 30 16163, Genoa, Italy.
Data accessibility	Repository name: Mendeley Data Data identification number: 10.17632/43s22jvj8w.1 Direct URL to data: https://data.mendeley.com/datasets/43s22jvj8w/1 Instructions for accessing these data: Merino, Danila; Bellassi, Paolo; Morelli, Lorenzo; Athanassiou, Athanassia (2023), “ANTIMICROBIAL, BARRIER, AND MECHANICAL PROPERTIES OF BIOCOMPOSITES PREPARED FROM CARROT POMACE AND WHEAT GLUTEN WITH VARIED EUGENOL CONTENT”, Mendeley Data, V1, doi: 10.17632/43s22jvj8w.1
Related research article	The related research article entitled “Blending of polysaccharide-based carrot pomace with vegetable proteins for biocomposites with optimized performance for food packaging applications” was published at Food Hydrocolloids. [1] DOI: 10.1016/j.foodhyd.2024.109903

## 1 Value of the Data

• These data are valuable as they offer insights into a novel composite biomaterial derived from carrot pomace, wheat gluten, and polyglycerol-3 plasticizer, enhanced with eugenol. This innovation holds potential for sustainable and functional food packaging materials, addressing both environmental concerns and food safety requirements.
• Researchers in food packaging, material science, and biotechnology can benefit from these data. The dataset bridges disciplines, offering antimicrobial, barrier, and mechanical properties information, inspiring collaborative efforts to develop sustainable, versatile and effective materials.
• Other researchers can reuse these data to advance their investigations on similar composite films. The dataset serves as a foundation for developing optimized formulations, tweaking eugenol content, and exploring other natural additives to fine-tune properties for specific applications.
• By leveraging the antimicrobial, barrier, and mechanical data, developers can expedite the prototyping of food packaging materials. This accelerates the transition from laboratory findings to practical applications, enhancing the efficiency of material design processes.
• Industries seeking eco-friendly packaging solutions can benefit from the dataset, especially nowadays that new EU regulations are expected from the ordinary legislative procedure that limit or prohibit the use of plastic single-use materials. It provides a baseline for evaluating the potential of bio-based materials, encouraging the adoption of sustainable practices, and contributing to the reduction of plastic waste.
• The dataset's antimicrobial and barrier properties information extends beyond food packaging. Medical and healthcare sectors can explore these data for the development of biocompatible materials for wound dressings, and other antimicrobial applications.

## 2 Background

The dataset presented in this 'Data in Brief' article supplements the research published under the title “Blending of polysaccharide-based carrot pomace with vegetable proteins for biocomposites with optimized performance for food packaging applications”. The primary focus of the associated article is the exploration of sustainable alternatives to petroleum-derived plastics for food packaging. The authors developed natural polymeric composite materials by blending carrot pomace (CP) with two vegetable proteins, wheat gluten (Gn) and zein (Z), at various ratios. The optimization process involved the addition of the plasticizer polyglycerol-3, resulting in improved mechanical and barrier properties. Notably, the developed materials lacked antimicrobial features.

To further enhance these materials, the best-performing formulation—comprising plasticized CP and 50 wt.% Gn blend—was combined with varying amounts of eugenol, which was chosen because of its natural origin and demonstrated antimicrobial properties when incorporated at low percentages. [2] This 'Data in Brief' article reports on the antimicrobial properties, complemented by assessments of mechanical strength and water vapor barrier properties, crucial for packaging applications. By presenting this dataset, we aim to contribute additional insights beyond the original research article, offering a more comprehensive understanding of the material properties and potential applications.

## 3 Data Description

The dataset published here supports an original research article [1] and is primarily divided into three main categories: Antimicrobial Properties, Barrier Properties, and Mechanical Properties. Each of these categories has its own file available at Mendeley Data [3].

### 3.1 Antimicrobial properties

**Fig. 1:**
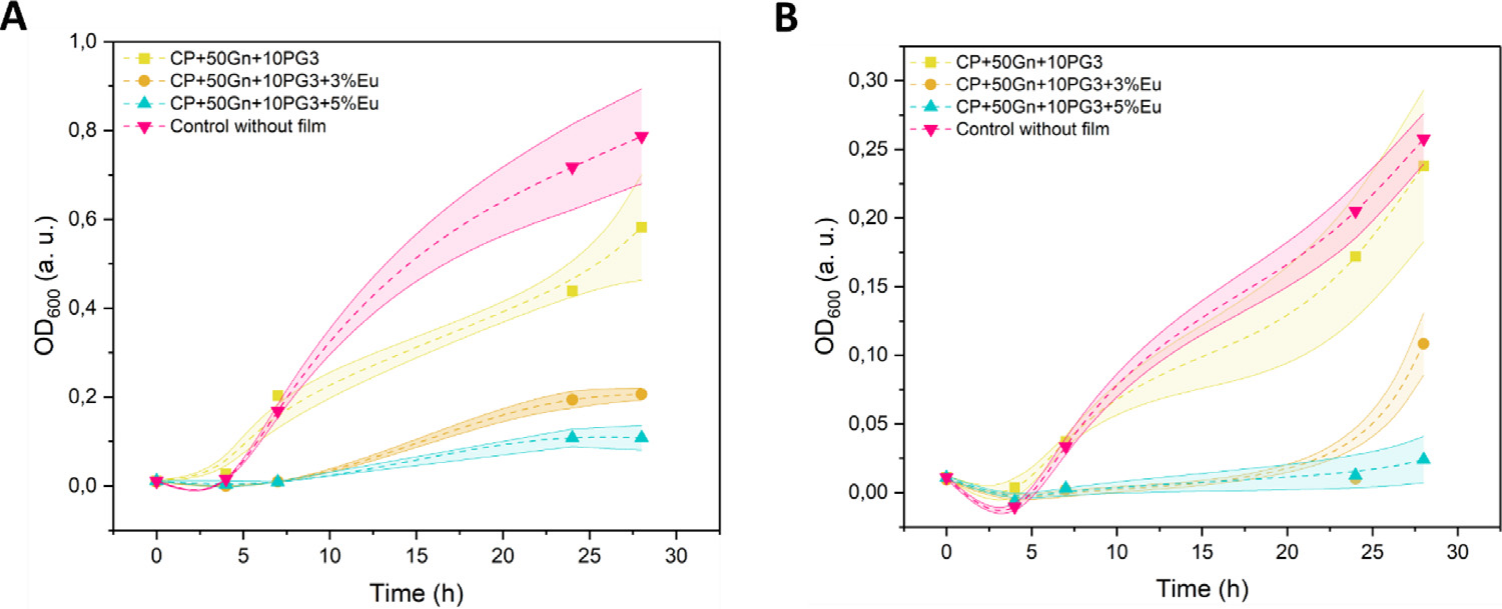
Antimicrobial properties. A: Optical density measured at 600 nm (OD_600_) vs time (h) for composites incubated with *E. coli* ATCC 25922. B: Optical density measured at 600 nm vs time (h) for composites incubated with *S. aureus* ATCC 6538.

The file entitled “Data-antimicrobial” contains antimicrobial properties of the composites evaluated in liquid media against the bacterial strains *Staphylococcus aureus* ATCC 6538 and *E. coli* ATCC 25922. Analyzed data are included in Fig. 1. Raw Data Files include the records of OD_600_ measurements for biocomposite films with various eugenol concentrations (0, 3, and 5 wt.%) during 28 h. Furthermore, controls encompassing cases without the film and without both the film and inoculum were also undertaken and are included in the data set. Three replicates were done, and the results were represented as mean ± Standard Deviation (SD).

### 3.2 Barrier properties

The file labeled ``Data-WVP'' encompasses essential water vapor permeability (WVP) raw and analyzed data. Within this file, two distinct tables can be observed, each serving a specific purpose.

**Fig. 2:**
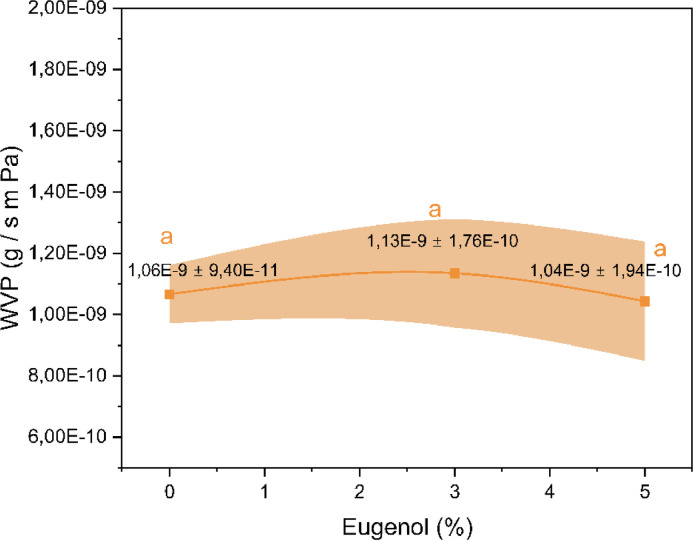
Water vapor permeability (g s^−1^m^−1^Pa^−1^) of biocomposites with 0, 3, and 5 wt.% eugenol.

The initial table provides insights into the experiment's timeline, providing data regarding the weight of the capsules at specific intervals. The subsequent table outlines the experimental conditions, delving into factors such as Relative Humidity (RH) within and outside the chambers, film thickness, the slope of the weight loss vs. time curves (obtained from the first table), films’ exposed surface area, water vapor pressure at the experimental temperature, as well as values pertaining to water vapor transmission rate (WVPTR) and WVP. The experimental procedure was conducted in triplicate for each developed biocomposite. This approach led to the generation of a comprehensive representation of results (depicted in Fig. 2), displaying the mean WVP values alongside their corresponding SD. Besides, data was further analyzed by one-way analysis of variance (ANOVA) and Tukey's test using Origin 2019b software. The same letters indicate the samples are not significantly different with 95 % confidence.

### 3.3 Mechanical properties

The file ``Data-Mechanical Properties'' contains comprehensive information regarding mechanical properties. It is divided into four distinct sheets. The initial sheet presents a table that has been extracted from the ``Bluehill Universal'' software. It includes the specimen label, Young's modulus, maximum load, tensile stress at yield (slope threshold 50%), tensile strain (extension) at yield (slope threshold 50%), tensile stress at maximum load, tensile strain (extension) at maximum load, area under the curve, and details on sample dimensions and crosshead speed. The subsequent three sheets encompass the raw data for each specimen, categorized by the corresponding eugenol content. Every sample underwent four separate analyses, and the data from these four replicates have been sequentially incorporated within each sheet.

**Fig. 3:**
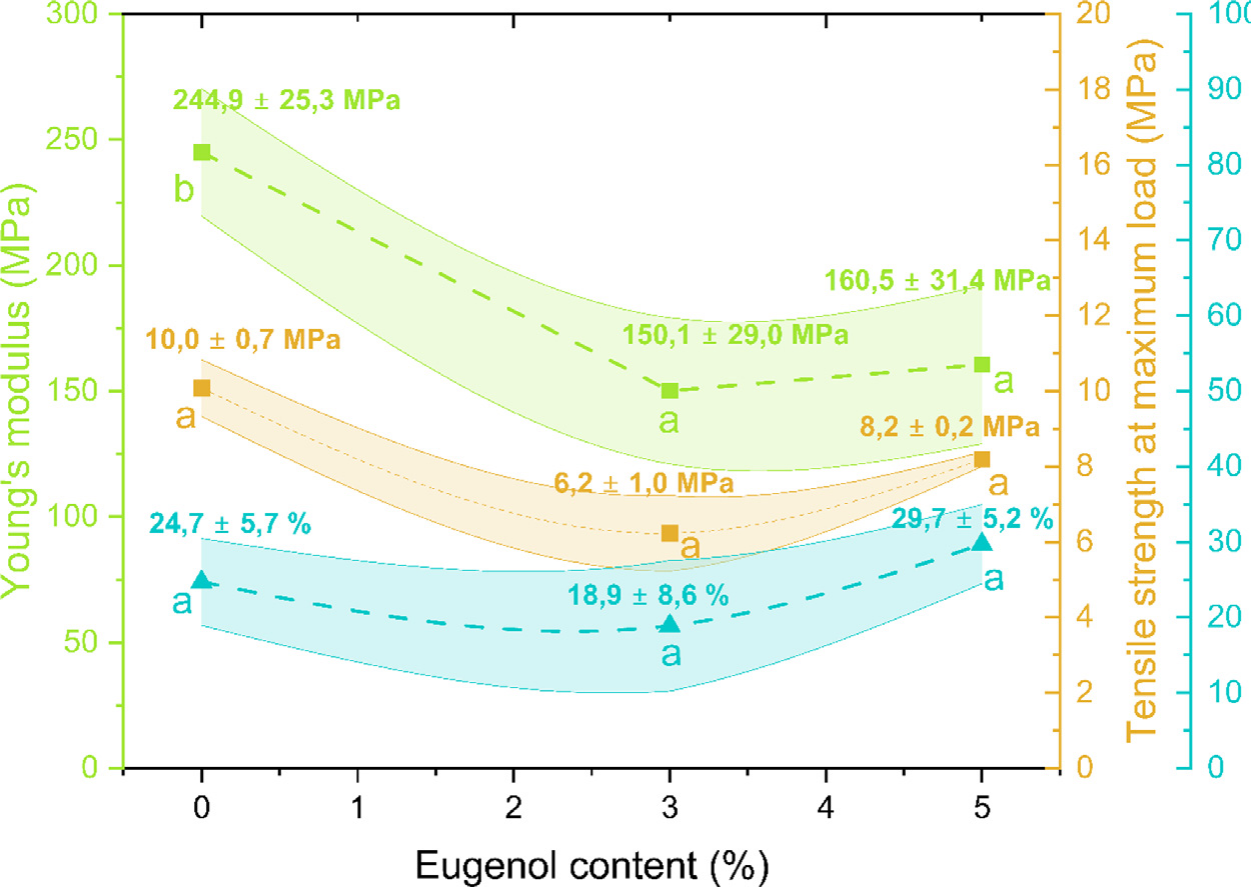
Mechanical properties of biocomposites. Letters of the same color on the top of the data points indicate whether the samples are significantly different (different letters) or not (equal letters) with 95 % confidence.

Data was subsequently analyzed in Origin 2019b and Young's modulus, tensile strength at maximum load, and elongation at break (%) are represented in Fig. 3 as mean ± SD. One-way analysis of variance (ANOVA) and Tukey's test were also performed and results are included in Fig. 3 with letters.

## 4 Experimental Design, Materials and Methods

### 4.1 Materials

Carrot pomace (CP), with a composition of 52 wt.% cellulose, 43 wt.% pectin, and 3 wt.% hemicellulose [4], was supplied by Harms Food (Zeven, Germany) as a dried powder with a particle size smaller than 50 µm and it was used as received. The plasticizer polyglycerol-3 (PG3) was generously provided by SPIGA Nord S. p. A. (Italy). As per the supplier's information, the product is plant based, is free from genetically modified organisms (GMOs), possesses Kosher and Halal certifications, and is authorized for the production of cosmetic and food additives. Wheat gluten (Gn), eugenol (Eu), and a solution of ammoniac (28.0–30.0 % NH_3_ basis) were purchased from Merck (Italy). Additionally, Milli-Q water was utilized throughout the experiments.

### 4.2 Bioplastics preparation

CP-Gn bioplastics containing 10 wt.% PG3 and varying amounts of Eu (0 wt.%, 3 wt.%, and 5 wt.%) were obtained through mixing and compression molding, following the technique outlined in our previously work [5]. Briefly, every 1 g of CP, 250 µL of MilliQ water were added as processing aid. Here, for the composites, same procedure was followed but instead of using water, a 1 M NH_3_ solution was used. Besides, in all cases ethanol was added to reach a concentration of 70:30 ethanol: water to favor gluten solubility [[6], [7], [8]]. The precise composition of each ingredient in the bioplastic is provided in Table 1.

**Table 1:** Chemical composition of bioplastics.

Name	CP (g)	Gn (g)	PG3 (g)	Eu (g)	NH_3_ 1 M (µL)	Ethanol (µL)
CP+50Gn+10PG3	0.45	0.45	0.1	–	112	373
CP+50Gn+10PG3+3 %Eu	0.43	0.43	0.1	0.03	107	356
CP+50Gn+10PG3+5 %Eu	0.42	0.42	0.1	0.05	105	350

To prepare the bioplastics, the initial step involved blending the protein, plasticizer, and antimicrobial agent in a mortar with pestle. This mixture was combined with an alkaline ethanolic solution, after which the CP powder was introduced. The components were thoroughly mixed until a consistent, uniform dough was achieved, reaching a softball-like consistency. Subsequently, the blends were subjected to hot pressing at 90 °C for 5 min under a pressure of 5 tons, before following a pre-heating step with a duration of 5 min at the same temperature, a procedure that lead to the evaporation of the solvents and the formation of the final films.

### 4.3 Bioplastics characterization

#### 4.3.1 Antimicrobial activity

A liquid test was conducted to evaluate the antimicrobial potential of the bioplastics against the food-pathogenic bacteria *Staphylococcus aureus* (*S. aureus* ATCC 6538, Gram-positive) and *E. coli* (*E. coli* ATCC 25922, Gram- negative). The strains were brought back to an active state from frozen stocks at – 80 °C using the Mueller Hinton Broth (MHB; Thermo Fisher Scientific™, Oxoid™, Waltham, MA, USA) medium. The overnight stock suspensions of *S. aureus* and *E. coli* were diluted to obtain a turbidity of 0.5 McFarland, corresponding to ∼ 1.5 × 10^8^ colony forming units per millilitre (CFU mL^−1^). The final inoculation was done to reach a final concentration of ∼ 10^6^ again, CFU mL^−1^.

The antimicrobial activity of the films was evaluated by monitoring the growth of microorganisms for 28 h of incubation in contact with the film [9]. Accordingly, three pieces of selected bioplastics (each piece with a surface area/volume ratio of 0.6 cm^2^ mL^−1^) were sterilized by UV-exposure for 30 min per face, afterwards immersed in 2 mL of MHB medium using a square septate plate (Sterilin™ 100 239 mm Square Petri Dishes), and microbial growth was monitored at the incubation temperature of 37 °C using the OD600 after a pre-incubation at 4 °C for 12 h [9]. One well without film was used as a positive control, and one without inoculum was utilized as a negative control. Tests were conducted in triplicate, and results were expressed as mean ± SD.

#### 4.3.2 Water vapor permeability (WVP)

**Fig. 4:**
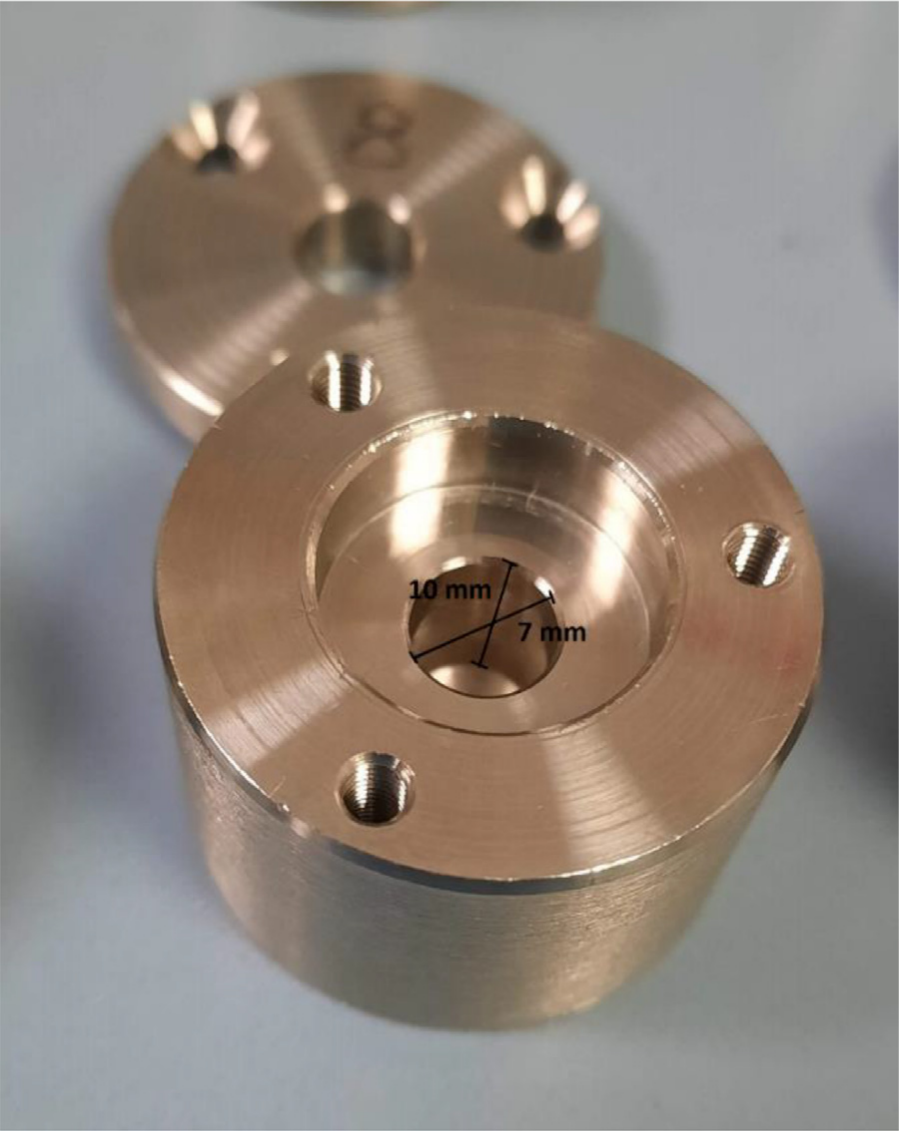
Photograph of a capsule used for measuring the WVP with 7 mm inside diameter and 10 mm inner depth.

The water vapor permeability (WVP) was determined using aluminum-based permeation capsules with a 7 mm inside diameter and a 10 mm inner depth (Fig. 4) filled with 300 µL of Milli-Q water (100 % RH) and sealed on the top with the biocomposite samples using two O-rings and a ring-shaped lid adjusted with screws. The capsules were stored in a chamber at 0 % RH simulated with dried silica gel. Their weight loss was checked over time and plotted. A linear fitting allowed the obtaining of the slope from the representation of capsules' weight vs. time. Then, this slope was divided by the exposed film area (m^2^) in order to obtain the water vapor transmission rate (WVTR). Later, these values were used in Eq. (1) to get the WVP (g m^−1^ s^−1^ Pa^−1^).

(1) $$WVP=WVTR\;\times t/\left(P_{H2O}\;\times \;\Delta RH\right)$$

where t is the average thickness of each sample (m), $P_{H2O}$ (Pa) is the water vapor saturation pressure at the test temperature (20 °C), and ∆RH is the difference in vapor pressure through the film. All samples were analyzed in triplicate, and results were expressed as average ± SD.

#### 4.3.3 Mechanical properties

An INSTRON 3365 machine (Instron^Ⓡ^, UK) was used to study the mechanical properties of biocomposites. Before being examined, the samples were conditioned in an environmental chamber (Espec SH-262, USA) for 48 h at 24 °C and 50 %RH and cut into a dog-bone shape, with a straight region measuring 25.01 mm length and 3.98 mm width. Afterwards, each specimen's thickness was determined with a digital micrometer having a 0.001 mm precision (Mitutoyo, USA). Four specimens from each sample were examined at a 3 mm/min drawing pace. Results were presented as mean ± standard deviation (SD) and included the sample's Young's modulus (MPa), tensile strength (MPa), and elongation at break (%) values.

## Limitations

None.

## Ethics Statement

The proposed data does not involve any human subjects, animal experiments, or data collected from social media platforms.

## CRediT authorship contribution statement

**Danila Merino:** Conceptualization, Methodology, Data curation, Formal analysis, Visualization, Writing – original draft. **Paolo Bellassi:** Data curation, Formal analysis. **Lorenzo Morelli:** Resources. **Athanassia Athanassiou:** Funding acquisition, Writing – review & editing.

## Data Availability

ANTIMICROBIAL, BARRIER, AND MECHANICAL PROPERTIES OF BIOCOMPOSITES PREPARED FROM CARROT POMACE AND WHEAT GLUTEN WITH VARIED EUGENOL CONTENT (Original data) (Mendeley Data).
